# A Rare Case of Hemophagocytic Lymphohistiocytosis Associated with Parvovirus B19 Infection

**DOI:** 10.7759/cureus.897

**Published:** 2016-11-24

**Authors:** Cai Yuan, FNU Asad-Ur-Rahman, Khalid Abusaada

**Affiliations:** 1 Internal Medicine Residency, Florida Hospital-Orlando; 2 Graduate Medical Education, Florida Hospital-Orlando

**Keywords:** hemophagocytic lymphohistiocytosis, parvovirus b 19, multi-organ failure

## Abstract

Hemophagocytic lymphohistiocytosis (HLH) is a rare but life-threatening syndrome resulting from excessive immune activation. Secondarily, HLH is often associated with autoimmune disease, infection, and malignancy. The most common infectious trigger is Epstein-Barr virus (EBV) infection. HLH is rarely triggered by parvovirus B19. We discuss a case of a 62-year-old male who presented with multi-organ failure with presumed septic shock who eventually was diagnosed with HLH, with positive parvovirus B19 deoxyribonucleic acid (DNA) polymerase chain reaction (PCR). Prompt treatment with dexamethasone resulted in significant clinical resolution.

## Introduction

Hemophagocytic lymphohistiocytosis (HLH) is a rare but life-threatening syndrome caused by uncontrolled immune activation leading to excessive macrophage activity and cytokine release resulting in tissue damage and multiple organ failure [1]. It was first recognized as a familial childhood immune dysregulatory disorder which was named “familial hemophagocytic reticulosis” in 1952 [2]. HLH is divided into primary or familial HLH and secondary HLH. Primary HLH is caused by a defect in the gene encoding the molecules in the pathway of cytotoxic T lymphocytes (CTL) and natural killer (NK) cells. Primary HLH is mainly seen in children, and the onset of familial HLH is usually at less than two years of age [3]. Secondary HLH occurs more commonly in adults. It is believed to be triggered by autoimmune disease, infection or malignancy [4]. The most common infectious trigger is Epstein-Barr virus (EBV) infection [5]. It is not commonly associated with parvovirus B19 infection. In a national survey in Japan, EBV was the most predominant cause among the 301 infection-associated HLH cases (163 cases, 54.2%), and only three cases (1.0%) were caused by parvovirus B19 [6].

Secondary HLH is often difficult to diagnose as the disease and laboratory findings are nonspecific. A high index of suspicion is needed for early diagnosis. It can mimic many other systemic inflammatory diseases including sepsis with multi-organ failure. If left untreated, the disease is generally fatal, with median survival estimated to be less than two months [7]. Early correct diagnosis of the disease with prompt effective treatment is crucial to improve mortality rates in this group of patients. Informed patient consent was obtained from the patient for this study.

## Case presentation

A 62-year-old African-American male with a past medical history of schizophrenia presented to the emergency department (ED) with two-day duration of progressive confusion. On presentation, the patient was lethargic, hypotensive with a blood pressure of 97/67 mmHg, pulse of 110/minute, respiratory rate of 23/minute and temperature of 99° F. He was hypoglycemic with a blood glucose level of 27 mg/dl. A physical examination revealed splenomegaly with cool peripheries without any bruising or purpura.

Initial laboratory studies revealed severe anemia with hemoglobin 4.4 g/dl, white cell count 12580/ ml (20% bands, 48% neutrophils, and 20% lymphocytes), platelet count 58,000/ml. International Normalized Ratio (INR) was 2.50, activated partial thromboplastin time (APTT) was 36.9 seconds. The metabolic panel was remarkable for severe metabolic acidosis with an anion gap of 34, serum creatinine (Cr) 2.43 mg/dl, alanine aminotransferase (ALT) 2029 U/L, aspartate aminotransferase (AST) 2267 U/L and lactate dehydrogenase (LDH) >2500 U/L. (Table 1)

**Table 1:** Trend of laboratory studies during hospitalization

Hospital Day	1	19	30
Aspartate aminotransferase (U/L)	2267	43	27
Alanine aminotransferase (U/L)	2029	69	28
Alkaline phosphatase (U/L)	257	198	146
Total bilirubin (mg/dL)	11.6	1.2	0.5
Albumin (g/dL)	3.7	3.6	3.1
International Normalized Ratio (INR)	2.5	1.25	
Blood urea nitrogen (mg/dL)	28	82	65
White blood cell count (K/mm3)	12.8	13.39	8.04
Hemoglobin (g/dL)	4.4	8.9	7.9
Platelets (K/mm3)	58	52	64
Ferritin (ng/mL)	32837	2347	2328
Creatinine (mg/dL)	2.43	3.23	3.65

A chest X-ray revealed bibasilar atelectasis. A computed tomography (CT) scan of the brain revealed no acute hemorrhage. Broad-spectrum antibiotics and multiple vasopressors were initiated for presumed septic shock from pneumonia. He developed worsening respiratory compromise necessitating endotracheal intubation and mechanical ventilation for hypoxemic respiratory failure. On hospital stay day 2, he had a cardiac arrest with pulseless electrical activity but was successfully resuscitated. The patient’s urine output deteriorated and serum creatinine trended up requiring the initiation of hemodialysis.

**Figure 1:**
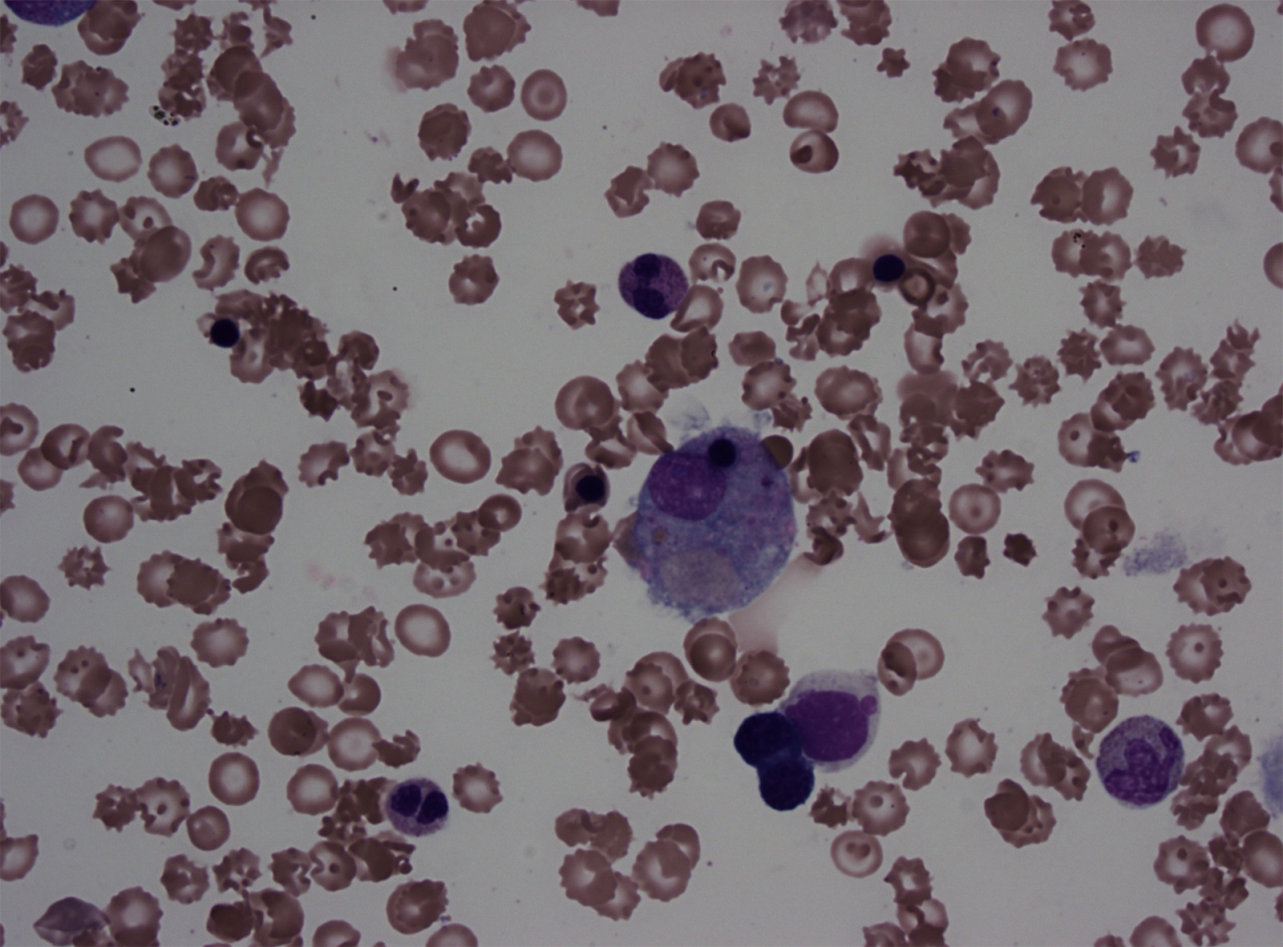
Bone marrow biopsy with hemophagocytic cells

Further infectious workup was negative for viral respiratory polymerase chain reaction (PCR), human immunodeficiency virus (HIV) serology, acute infectious hepatitis, cytomegalovirus PCR, and herpes simplex virus (HSV). Epstein-Barr virus (EBV) immunoglobulin G (IgG) antibody was positive. A transthoracic echocardiogram (TTE) was negative for vegetations. The serum parvovirus B19 DNA PCR returned as positive. A CT abdomen revealed splenomegaly and ground glass opacification of both the lung bases. Serum and urine electrophoresis were negative. Serum ADAMTS13 (a disintegrin and metalloproteinase with a thrombospondin type 1 motif, member 13) and antinuclear antibodies (ANA) were negative. The continued evaluation revealed a significant elevation of ferritin levels of 32,837 ng/ml (normal range 24-336 ng/ml), raising the concern for HLH. Additional laboratory data revealed that the fibrinogen level was 190 mg/dl (range 188-436 mg/dl) and fibrinogen split products were 640-1280 mcg/dl. The serum triglyceride level was 92 mg/dl and NK cell activity was within normal limits. A bone marrow biopsy was performed, given the concern for HLH, which demonstrated hypercellular bone marrow with erythroid hyperplasia and rare hemophagocytic cells. (Figure 1)

The soluble CD25 level was elevated to 2740 u/ml (normal range < 1033 u/ml). Thus our patient fulfilled five out of eight clinical criteria for HLH (splenomegaly, cytopenia involving at least two cell lines, high serum ferritin, high soluble CD25 level and evidence of hemophagocytic cells on bone marrow). (Table 2)

**Table 2:** Criteria for hemophagocytic lymphohistiocytosis diagnosis based on the 2004 trial [3]

Five of eight clinical criteria must be present for diagnosis
1. Fever (>38.5°C)
Splenomegaly
Pancytopenia (at least two cell lineages must be affected)
Hemoglobin <9 g/mL
Platelets <100 K/mm3
Neutrophils <1 × 109 L/min
Hypertriglyceridemia (265 mg/100 mL or more) and/or hypofibrinogenemia (150 mg/100 mL or less)
Ferritin 500 ng/mL or more
Hemophagocytosis in the bone marrow, spleen, or lymph nodes
Low or absent NK (natural killer) cell activity
Elevated soluble IL-2 receptor (>2400 U/mL) or >2 standard deviations above the age-adjusted mean

This prompted the initiation of dexamethasone which led to significant clinical improvement. Etoposide was not given due to underlying severe liver dysfunction. The patient was extubated and steroids were gradually tapered per protocol. His liver enzymes and coagulation studies gradually normalized. His platelet count and ferritin level improved gradually. However, the patient continued to require renal replacement therapy upon discharge. The patient was discharged home after 31 days of hospitalization. Six months later on follow-up, the patient was taken off hemodialysis due to significant improvement in renal function with a Cr level of 1.68 mg/dl, with a platelet count of 294 x 10 3/mL and a ferritin level of 928 ng/ml.

## Discussion

HLH is a rare disorder with high mortality. It is estimated that the median survival is less than two months if left untreated, with an overall mortality of 58% to 75% in adults [1, 7]. It is important to note that the prognosis is especially dismal in patients with underlying malignancy. Diagnosis and prompt treatment are essential to improve the patient’s outcome. The true incidence and prevalence of HLH in adults remain unknown due to probable underdiagnosis [8]. The diagnosis of HLH is often missed as its clinical presentation is extremely unspecific. It can mimic system inflammatory response syndrome from underlying infection or hematopoietic malignancy and is often treated as septic shock. Our patient presented with altered mental status with multi-organ failure on initial workup. He was started on broad-spectrum antibiotics for possible underlying infection without significant improvement, until he received dexamethasone for presumed HLH. As our case elucidated, a high index of suspicion is needed for prompt diagnosis of HLH. In many cases, simultaneous treatment for HLH and sepsis are required while waiting for final lab results.

The infectious trigger for secondary HLH most commonly seen is EBV infection [5]. It is rare to be associated with parvovirus B19. Our patient had positive EBV immunoglobulin G (IgG) antibody, but negative immunoglobulin M (IgM) antibody, which excluded the possibility of being previously triggered by EB virus. The parvovirus B19 deoxyribonucleic acid (DNA) polymerase chain reaction (PCR), however, was positive. The exact pathogenesis of secondary HLH remains unknown. One hypothesized mechanism is that the infectious viral protein of the trigger inappropriately activates cytotoxic and helper T cells, which leads to excessive and uncontrolled cytokine release resulting in tissue damage [4, 7]. An additional hypothesis is that defective T cells and NK cells fail to properly remove antigen resulting in ongoing uncontrolled macrophagic activity [4, 7]. Soluble interleukin-2 receptor (soluble CD25) reflects the activation of T cells and NK cell activity. Its measurement was included into the diagnostic criteria of HLH in the 2004 guidelines [3].

Ferritin is an intracellular protein that plays an important role in iron storage. It is also a biomarker for inflammation and is elevated in many inflammatory states. The cutoff level for diagnosis of HLH was proposed to be greater than 500 ng/ml in the 2004 guidelines [3]. One of the clues to the diagnosis in our case was the extremely high ferritin level of over 32,000. It was reported that ferritin levels > 10,000 ug/L had 90% sensitivity and 96% specificity for HLH in a 2008 pediatric study [9]. It has been also shown that higher ferritin level at the time of diagnosis is associated with poorer prognosis and higher mortality [1-4]. It is therefore used as a prognostic factor to monitor the treatment effects. Our patient had extremely high levels of ferritin on initial workup. The ferritin level rapidly decreased with effective treatment, which presented a favorable prognosis.

Bone marrow biopsy is a common tool in the workup of cytopenia that many HLH patients may present. The detection of hemophagocytosis in the bone marrow is supportive of the diagnosis. However, it is not pathognomonic for HLH. It can be seen in a few other diseases including viral infections, especially EBV, and hematologic malignancies such as non-Hodgkin’s-lymphoma [10]. Its presence is estimated to have a sensitivity of 80% to 83% and a specificity of only 60% to diagnose HLH [4]. Therefore, its absence cannot rule out HLH and its presence alone is not sufficient for diagnosis.

The current consensus for the treatment of HLH consists of dexamethasone and etoposide. These agents are used due to their ability to target underlying activated immune cells and decrease cytokine release. Our patient did not receive etoposide due to significant liver failure. He did respond favorably to dexamethasone alone.

## Conclusions

Secondary hemophagocytic lymphohistiocytosis (HLH) is a rare but often fatal disease. Clinicians should have a high index of suspicion of HLH when encountering a case of septic shock with multiple organ failures, especially if a source of sepsis is not identified. Ferritin level can be used in the initial diagnostic workup. A high level of ferritin should lead to further laboratory evaluation. Early diagnosis and prompt treatment are the key to improving survival in this patient group.
